# The Cholera and the Board of Health

**Published:** 1885-01

**Authors:** 


### THE CHOLERA AND THE BOARD OF HEALTH.

There seems to be a reasonable probability that the cholera will reach our shores during the coming spring. It is said that it has never prevailed in France epidemically, unless it has soon afterwards appeared in the United States.

With the prospects before us in the nature of even a possible invasion of this formidable malady, it is the most solemn duty of all the authorities, National, State and Municipal, which are in any sense custodians of the health and sanitation of the people, to afford every protection to the citizen which science, energy and money can command. The responsibilities of health boards were never so great before, for the reason that the laws of prevention, as relating to infectious or contagious diseases, were never so well understood as at the present time; consequently they could never be applied with so near an approach to certainty of success, as now.

The municipal or local Board of Health is not only charged with the immediate execution of all laws and orders handed down to it from higher authority, but it can and must create ordinances and orders itself on all matters pertaining to municipal hygiene, sanitation, and health. The statutes confer adequate power upon these Boards to meet every emergency, and the people will hold them responsible for the faithful and zealous execution of their trust.

Petty politics, parsimony, and individual preference must stand aside, in the presence of a great public danger, and "the greatest good to the greatest number," should be the idea first and foremost in every thought and act of the members of Boards of Health at this time. *Now* is the time for them to exercise their plenary powers, to the end that preventive measures may be taken in season. It will not serve to carry out these measures in a half-hearted or perfunctory manner; it must be done with zeal, energy, and dispatch, with no particular reference to the individual, but with sole reference to the whole body-politic.

In this connection we are pleased to note that the Buffalo Board of Health has already taken some steps in the direction named above by appointing a sanitary engineer and three sanitary inspectors, the latter being physicians. These are, we believe, thoroughly competent officers, and we have no doubt they will perform their duties conscientiously, promptly, and with a thoroughness commensurate with the high responsibilities resting upon them. Their duties, if we understand aright, are advisory in character, and it rests with the Board to see their recommendations carried out. Let this be done in a prompt and complete manner, and we have every confidence that great benefit will result from their labors.

Meanwhile the citizens, themselves, have a plain duty in this matter. It is to strengthen the hands of the Board of Health, and its officials, to the end that there shall be no captious criticism of methods, nor unnecessary delay in the correction of evils. Buffalo is already the third city in the State in size, and its population is rapidly increasing. It has outgrown the old methods which applied to its earlier days, relating to the internal administration of its affairs. These may have been well enough then but are too slow and incomprehensive for its present necessity. There should be a most complete and comprehensive examination of all dwellings (private residences as well as tenement houses), public buildings, manufactories and streets, to the end that everything pertaining to plumbing, drainage, water supply, ventilation, and cleanliness shall be put upon the highest plane of sanitary efficiency. If more inspectors are needed, as doubtless there will be, they should be appointed to meet the demands of the service, as they arise, and this without cavil or niggardly complaint. That they should be men skilled in sanitary science goes without saying.

Buffalo, from its natural position, should stand in the front rank of large cities, as regards the health of its inhabitants and its low death rate. Its atmosphere is invigorating, pure, and wholesome, and its climate has been pronounced equal to any in the world for the promotion of health, longevity, and both physical and mental vigor. If it has been rendered otherwise from artificial causes, these must be swept away by the considerate and timely action of the health authorities; and it is the first and bounden duty of every citizen to hold up the hands of these officials, to the end that our city may be placed in the best condition to resist the threatened invasion of cholera that sanitary science can possibly devise. This is but the reasonable demand of the civilization of to-day, and no intelligent citizen will be satisfied with anything less than the fullest protection which the State is seeking to provide.

